# Effects of Scorpion venom peptide B5 on hematopoietic recovery in irradiated mice and the primary mechanisms

**DOI:** 10.1038/srep15363

**Published:** 2015-10-20

**Authors:** Caixia Wang, Meixun Zhou, Ting Li, Yan Wang, Baiqian Xing, Tianhan Kong, Weihua Dong

**Affiliations:** 1 Department of Hematology, Guangzhou First People’s Hospital, Guangzhou Medical university, Guangzhou, Guangdong 510182, PR China; 2 Department of Pathophysiology, Guangzhou Medical University, Guangzhou, Guangdong 510182, PR China

## Abstract

Scorpion venom peptide B5 (SVP-B5) stimulates recovery of hematopoiesis after exposure to radiation. However, its radioprotective effects and mechanisms are still unclear. The aim of this study was to investigate the effects of SVP-B5 on hematopoietic recovery in mice after total body irradiation (TBI) at a dose of 7.5Gy and 6Gy and to explore the possible primary mechanisms. SVP-B5 at a dose of 2.63 μg/kg significantly reduced the mortality rate of mice after TBI (p < 0.05). It showed markedly protective effects against radiation injury. SVP-B5 also significantly increased the number of bone marrow nucleated cells (BMNCs) and increased the colony forming unit (CFU) number in irradiated mice, accelerated the post-irradiation recovery of peripheral blood leukocytes and platelets in mice. SVP-B5 treatment markedly reduced the Reactive Oxygen Species (ROS) levels in BMNCs after TBI, reduced γH2AX levels, and decreased the relative expression levels of p16 and p21 mRNA at day14 (d14) after irradiation. Our study indicated that SVP-B5 could partially mitigate radiation-induced DNA damage, enhance the post-radiation hematopoietic recovery, and improve the survival rate probably through the ROS-p16/p21 pathway.

Currently, more than 50 percent of the patients with malignant tumors require radiation therapy^1^. Exposure to ionizing radiation (IR) causes damage to DNA, protein, and lipids, as well as increase generation of reactive oxygen species (ROS)^2^. The toxicity of high-dose ionizing radiation (IR) is associated with induction of acute radiation syndromes involving the hematopoietic system^3^ and gastrointestinal tract^4^. Bone marrow (BM) suppression is the primary side effect of TBI of conventional cancer therapy. Acute and residual (or long-term) BM injury not only limits the success of cancer treatment but also adversely affects the quality of life of cancer patients after irradiation. Therefore, developing drugs that can promote post-irradiation hematopoietic recovery is of great importance for improving the therapeutic effects and provide radiation protection to patients with tumors.

Scorpion venom peptide (SVP) is an active ingredient that has been isolated from the scorpion venom of *Buthus martensii* Karsch by our research lab. Previous studies have shown that, in mice, SVP increases the levels of several cytokines (SCF, IL-1α, IL-6, and GM-CSF) after irradiation, promotes the formation of splenic nodules in irradiated mice, significantly increases the number of bone marrow nucleated cells (BMNCs) and the bone marrow cell proliferation index, increases the numbers of hematopoietic stem/progenitor cells such as the colony forming unit-spleen (CFU-S) and colony forming unit of granulocyte-macrophage (CFU-GM), and does not cause any apparent side effects in mice^5^. The cell proliferation-promoting effects of SVP might be related to its activation of the JAK/STAT3 signal transduction pathway^6^. Scorpion venom peptide II (SVPII) is one of the peptide components that are isolated from the scorpion venom of *Buthus martensii* Karsch. SVPII can promote BMNCs colony formation and the proliferation of postradiation M-NSF-60 cells. It has a synergistic effect with interleukin-3 (IL-3) and can significantly upregulate the IL-3 receptor expression in M-NSF-60 cells after irradiation^7^.

SVP-B5 is a small peptide with a molecular weight of 7212.33 Da, isolated and purified from SVPII. Its complete amino acid sequence is VRDGYIADDK NCAYFCGRNA YCDDECKKNG AESGYCQQAG VYYNACWCYY LLDDVVIIIP SGCDQW^8^. Preliminary studies found that SVP-B5 could promote the proliferation of rat bone marrow-derived mesenchymal stem cells (rBMSCs) and could inhibite the increase of Senescent cells in bone marrow-derived mesenchymal stem cells (MSCs) after irradiation. Histone was identified as the possible molecular target of SVP-B5 in the regulation of rBMSC proliferation^9^,^10^. This study proposed to observe the effects of SVP-B5 on bone marrow hematopoietic recovery and survival in mice after irradiation. We further explored the possible primary mechanism of SVP-B5 on promoting hematopoietic recovery of the bone marrow and repairing radiation-induced injury.

## Results

### SVP-B5 prolonged the survival time and improved the survival rate of mice after TBI

**Figure 1:**
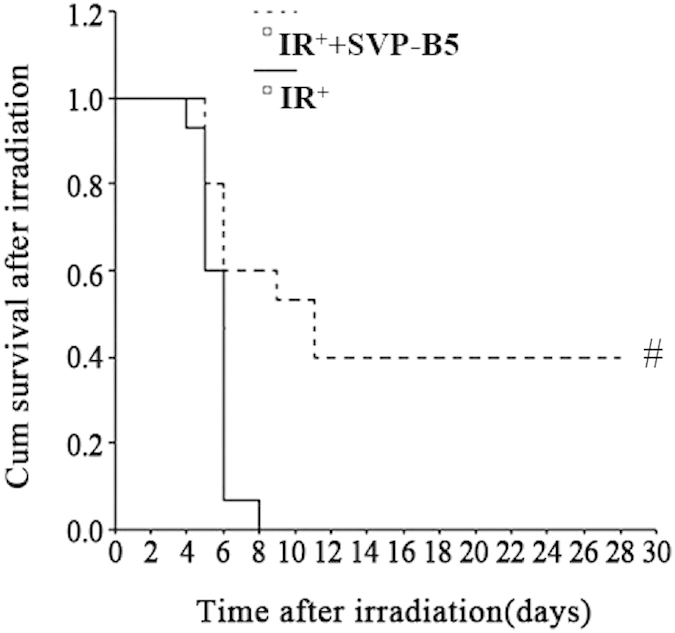
Effects of SVP-B5 on the survival rate of mice after TBI. Mice underwent intraperitoneal injection of SVP-B5 (2.63 μg/kg) (IR^+^+SVP-B5 group) or equal amounts of saline (IR^+^ group) 2h before TBI and once every other day after TBI (7.5Gy) until day 14. Effect of SVP-B5 on the survival rate of mice after TBI was observed (n = 15/group). # indicated a significant difference between the IR^+^ group and the IR^+^+SVP-B5 group, P < 0.05.

The survival test showed that all C57BL/6 mice in the IR^+^ group died within 8 days and the survival rate was 0. The average survival time was 6.67d. In the IR^+^+SVP-B5 group, 40% of the mice (6/15) survived until 30d after TBI, and the survival rate of mice was significantly improved as compared to that in the IR^+^ group. The average survival time of the mice also increased from 6.67d in the IR^+^ group to 14.9d in the IR^+^+SVP-B5 group (Fig. 1, Table 1). SVP-B5 significantly reduced the mortality of mice exposed to lethal TBI (*P* < 0.05) and prolonged the survival time of mice after TBI (*P* < 0.05), demonstrating protection against harmful radiation effects.

### SVP-B5 increased the bone marrow CFUs in irradiated mice

**Figure 2:**
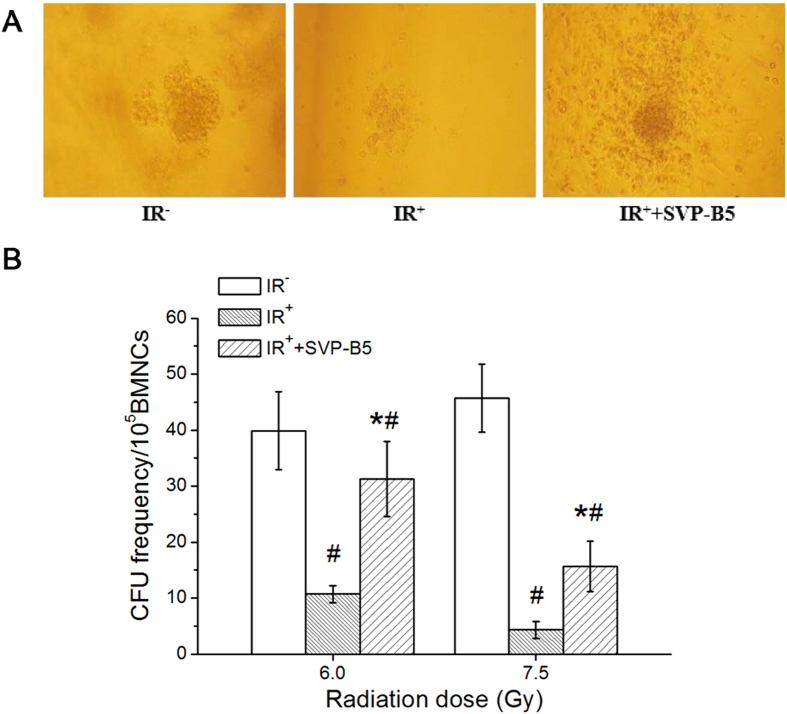
SVP-B5 promotes the colony formation of bone marrow hematopoietic stem cells in mice after irradiation. Semisolid cultures of BMNCs (2 × 10^5^/mL) of mice were performed after irradiation (6Gy or 7.5Gy). At 14d after culture, the effects of SVP-B5 on bone marrow hematopoietic stem cell CFUs were observed. (**A**) Morphology of bone marrow cells in various groups during the CFU assay (100×); (**b**) The number of CFUs was counted at 14d after culture and expressed as the number of CFUs/10^5^ BMNCs. The experiment was repeated three times, and the data were expressed as mean ± standard deviation. * Indicated a significant difference between the IR^+^+SVP-B5 group and IR^+^ group, P < 0.05; # indicated a significant difference between the IR^−^ group and the IR^+^+SVP-B5 group as well as the IR^+^ group, P < 0.05.

The hematopoietic progenitor cell culture showed that SVP-B5 increased the bone marrow CFU in irradiated mice (Fig. 2). In the IR^−^ group, the number of bone marrow CFUs was 45.7 ± 6.1 units/10^5^ BMNCs. In the IR^+^ group, the number of CFUs at a dose of 6Gy and 7.5Gy 14d after TBI were 10.7 ± 1.5 units/10^5^ BMNCs and 4.3 ± 1.8 units/10^5^ BMNCs, which was significantly lower than in the IR^−^ group (p < 0.05). SVP-B5 treatment promoted the colony-forming ability of BMNCs and the number of CFUs was 31.3 ± 6.7 units/10^5^ BMNCs and 15.7 ± 4.5 units/10^5^ BMNCs, respectively. Compared with the IR^+^ group, SVP-B5 treatment significantly improved the CFU-forming ability of the bone marrow after TBI (p < 0.05). The results suggested that SVP-B5 can partially protect the BMNCs against the harmful effects of radiation and partially promote the recovery and proliferation of hematopoietic progenitor cells in mice.

### SVP-B5 promoted hematopoietic recovery after TBI

**Figure 3:**
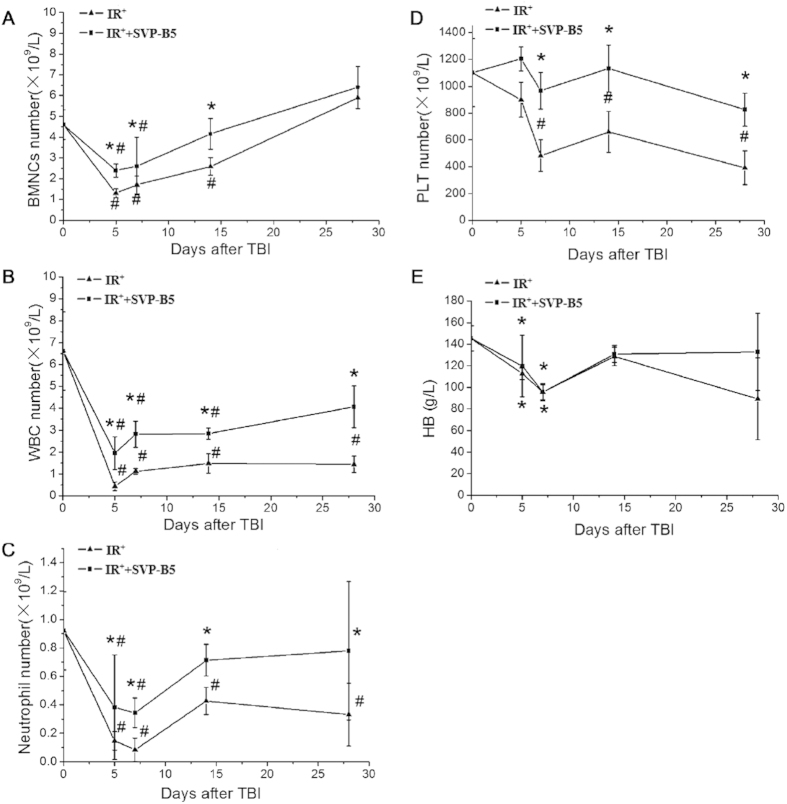
SVP-B5 promoted bone marrow and peripheral blood cell recovery in mice after TBI. Mice underwent intraperitoneal injection of SVP-B5 (2.63 μg/kg) or equal amounts of saline before TBI and once every other day after TBI (6Gy) until day 14. BMNCs and peripheral blood were collected before TBI and 5d, 7d, 14d, and 28d after TBI. SVP-B5 significantly promoted the recovery of the BMNC counts (**A**), white blood cell counts (**B**), neutrophil counts (**C**), platelet counts (**D**), and hemoglobin levels (**E**). The experiment was repeated three times, n = 9 per group at each time point. *P < 0.05 vs. IR^+^ group; # indicated a significant difference before and after TBI in the IR^+^ +SVP-B5 group and IR^+^ group, P < 0.05.

The number of BMNCs declined obviously after TBI. But in IR^+^+SVP-B5 group, BMNCs increased significantly at 5, 7, and 14 days compared with those in the IR^+^ group (*P* < 0.05). At 14d after TBI, the number of BMNCs increased and was 90.4% of the number before TBI in the IR^+^+SVP-B5 group, whereas the IR^+^ group only recovered to a level that was 56.4% (Fig. 3A). The white blood cell (WBC) count in the IR^+^+SVP-B5 group was higher than that in the IR^+^ group (*P*** **< 0.05) (Fig. 3B). SVP-B5 could significantly promote the recovery of neutrophils after TBI, and the neutrophil count in the IR^+^+SVP-B5 group was higher than that in the IR^+^ group at various time points during the study (*P* < 0.05) (Fig. 3C). After TBI, the platelet count reached the valley at 7d in both groups, dropped to a level that was 43.7% and 87.8% of that before TBI in the IR^+^ and IR^+^+SVP-B5 groups, respectively. At 14d after TBI, the platelet count in the IR^+^+SVP-B5 group recovered to the pre-TBI levels, but in the IR^+^ group, it only recovered to a level that was 59.9% (Fig. 3D). However, SVP-B5 did not have an effect on the post-TBI hemoglobin recovery (Fig. 3E).

### SVP-B5 inhibited the TBI-induced oxidative stress in BMNCs

**Figure 4:**
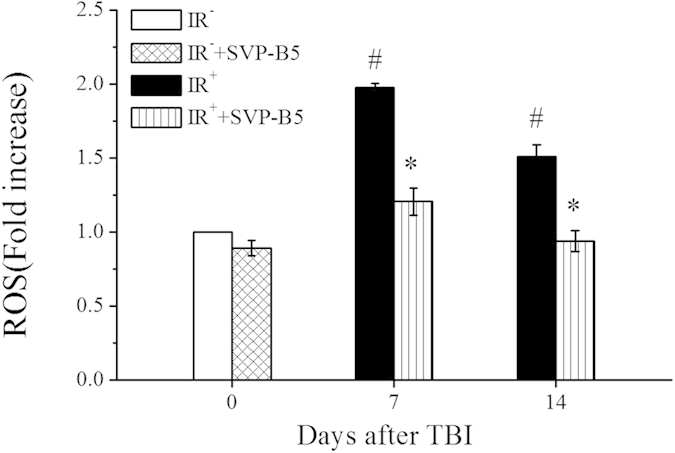
SVP-B5 inhibited the TBI-induced ROS production in BMNCs. Mice underwent intraperitoneal injection of SVP-B5 (2.63 μg/kg) or equal amounts of saline before and after TBI. The ROS levels in the BMNCs were detected by flow cytometry 7d and 14d after TBI, and compared with those in the BMNCs in non-irradiated mice (IR^–^ group). The experiment was repeated three times, n = 9 per group at each time point. *P < 0.05 vs. IR^+^ group; # indicated a significant difference between the IR^−^ group and the IR^−^ +SVP-B5 groups vs. IR^+^ group, *P* < 0.05.

As shown in Fig. 4, the ROS levels of BMNCs at 7d and 14d after TBI were increased compared with those in the IR^−^ group, and they were 2.00 and 1.50 times higher, respectively, than those in the IR^−^ group. SVP-B5 could slightly decrease the ROS levels in the non-irradiation mice, but there is no difference (*P* > 0.05). In the IR^+^+SVP-B5 group, the ROS levels at 7d and 14d after TBI were 1.20 times and 0.94 times higher, respectively, than those in the IR^−^ group. Compared with the IR^+^ group, the ROS levels in the IR^+^+SVP-B5 group, decreased significantly (*P* < 0.05). At 14d after TBI, the ROS level in the BMNCs returned to normal. The results showed that SVP-B5 could reduce ROS production and inhibit TBI-induced oxidative stress in the hematopoietic system.

### SVP-B5 mitigated TBI-induced DNA damage of BMNCs in mice

**Figure 5:**
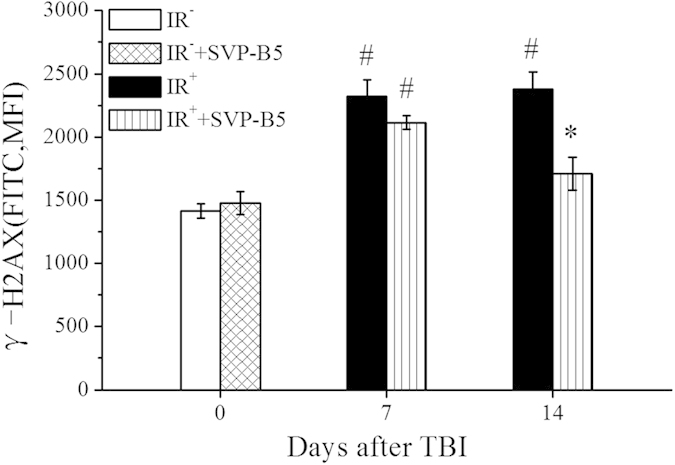
SVP-B5 reduced TBI-induced DNA injury in mice BMNCs. Mice underwent intraperitoneal injection of SVP-B5 (2.63 μg/kg) or equal amounts of saline before and after TBI. BMNCs were collected from non-irradiated mice and mice 7d and 14d after TBI. The γH2AX level was detected using flow cytometry. The experiment was repeated three times, n = 9 per group at each time point. *P < 0.05 vs. IR^+^ group; # indicated a significant difference between the IR^−^ group and the IR^−^ +SVP-B5 group vs. IR^+^ +SVP-B5 group and the IR^+^ group, P < 0.05.

At 7d after TBI, the mean fluorescence intensities (MFI) of the intranuclear γH2AX in the IR^+^ and IR^+^+SVP-B5 groups were 2320.00 ± 132.011 and 2115.00 ± 52.85, respectively, higher than IR^−^ group, and these values were significantly different from the IR^−^ group (*P* < 0.05). At 14d after TBI, the MFI in the IR^+^ group was 2376.25 ± 140.54, higher than that before TBI, and there were no significant differences at 7d and 14d after TBI (*P* > 0.05). However, the MFI in IR^+^+SVP-B5 group decreased to a level which was 1710.25 ± 131.64 at 14d after TBI and was significantly different from the value in the IR^+^ group (*P* < 0.05) (Fig. 5). The aforementioned results showed that SVP-B5 could reduce the TBI-induced elevation in the γH2AX levels of BMNCs and promote DNA damage repair.

### Effects of SVP-B5 on the relative mRNA expressions of p16^Ink4a^ and p21^Cip1/Waf1^

**Figure 6:**
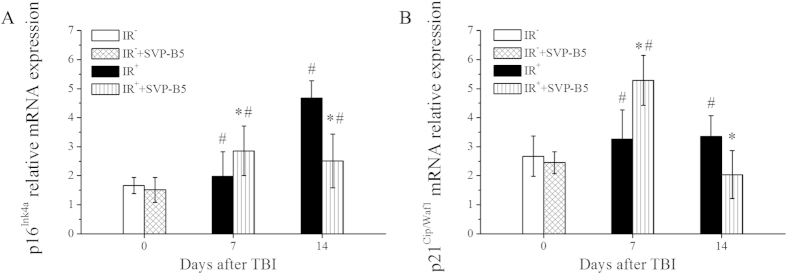
Effects of SVP-B5 on the relative expression levels of p16^Ink4a^ and p21^Cip1/Waf1^ mRNA Mice. underwent intraperitoneal injection of SVP-B5 (2.63 μg/kg) or equal amounts of saline before and after TBI. BMNCs were collected before TBI and 7d and 14d after TBI. The QT-PCR assay was used to detect the relative expressions of p16^Ink4a^ and p21^Cip1/Waf1^ mRNA in the nuclei. The ratio of the target gene mRNA content to GAPDH was used to assess expression levels. The experiment was repeated three times, n = 9 per group at each time point. *P < 0.05 vs. IR^+^ group; # indicated a significant difference between the IR^−^ group and the IR^−^ +SVP-B5 group vs. IR^+^ +SVP-B5 group and the IR^+^ group, P < 0.05.

Real-time quantitative PCR results showed that the relative expression of p16^**Ink4a**^ mRNA from BMNCs was 1.98 ± 0.84 at 7d after TBI, increased obviously compared with the pre-TBI levels (1.67 ± 0.28), especially in the IR^+^+SVP-B5 group which was 2.86 ± 0.85. The relative expression of p16^**Ink4a**^mRNA in the IR^+^ group remained high (4.68 ± 0.59) at 14 d after TBI, but declined to normal levels in the IR^**+**^+SVP-B5 group (2.50 ± 0.93) at 14d after TBI. There was a significant difference in the relative expression levels of p16^**Ink4a**^mRNA between the IR^+^ and the IR^**+**^+SVP-B5 groups at 14d after TBI (*P* < 0.05). The relative expression of p21^**Cip1/Waf1**^mRNA had similar changes as that of p16^**Ink4a**^ mRNA at 7d and 14d. In the IR^**+**^+  SVP-B5 group, the expression level of p21^**Cip1/Waf1**^mRNA first increased at 7d, followed by a decline at 14d. In the IR^+^ group, the expression level of p21^**Cip1/Waf1**^mRNA remained high at 14d after TBI. There was a significant difference in the relative expression level of p21^**Cip1/Waf1**^mRNA between the IR^+^ and the IR^**+**^+  SVP-B5 groups at 14d after TBI (*P* < 0.05, Fig. 6).

## Discussion

Radiation injury refers to acute, delayed, or chronic damage to body tissues caused by ionizing radiation (IR). IR can result in damage to cellular DNA, proteins, and lipids, leading to mitochondrion-dependent ROS increase, cell cycle arrest, apoptosis, and cellular senescence. Ultimately, it can lead to bone marrow suppression, radiation enteritis and damage in other organs. Although radiation injury has been fully proven in clinical practice and its mechanism has been gradually understood, effective prevention and treatment of radiation injury awaits further in-depth studies.

Scorpion venom is a biologically active substance with complex composition and a variety of physiological and pharmacological effects. Our lab isolated SVP and SVP II from scorpion venom and both of them have radiation-protective effects. SVP-B5 is a new polypeptide that was isolated and purified by our lab from SVP II. Our previous study showed that the toxicity of SVP-B5 was low (its LD_50_ was 0.105 mg/kg), and 1/40 LD_50_ (2.625 μg/kg) could effectively extend the life of mice after TBI and improve their survival rate from 0 to 40%, the median survival time was extended from 5 to 10 days, and the average survival time in the IR^+^ group was extended from 6.67 days to 14.9 days, indicating that SVP-B5 has significant radiation-protective effects.

The hematopoietic system is one of the most sensitive tissues to radiation. IR may easily cause bone marrow suppression, thereby resulting in infection, anemia, or bleeding. Bone marrow suppression is the leading cause of death in those patients^11^. SVP-B5 helped increase the number of BMNCs. At 14d after TBI, the number of BMNCs returned to the levels before irradiation, but remained low in the IR^+^ group and was significantly lower than that in the SVP-B5 treatment group. Bone marrow is the main organ of hematopoiesis, and the recovery of its nucleated cells is of significant importance for the recovery of the hemogram. In the hemogram, changes in the numbers of white blood cells, granulocytes and platelets are basically consistent with those of BMNCs. SVP-B5 promoted proliferation of clonal subpopulation of bone marrow progenitor cells after ionizing radiation and thus increased the number of BMNCs.SVP-B5 could significantly increase the number of white blood cells, neutrophils and platelets in mice after TBI, which was of significant importance for reducing the rate and severity of infections and deaths caused by hemorrhage.

The most important reason for the radiation injury is excessive ROS production in cells, which leads to subsequent DNA damage, cell cycle arrest, apoptosis, and stress-related reactions^12^. The intracellular ROS levels were elevated after TBI, causing oxidative stress in HSCs, increased DNA damage, and long-term bone marrow suppression^13^. Antioxidants play an important role in the treatment of radiation injury caused by elevated ROS^14^,^15^. SVP-B5 treatment could reduce the post-TBI ROS levels as an antioxidant and might help alleviate radiation-induced oxidative stress, thereby reducing hematopoietic system damage and promoting hematopoietic recovery. These results were consistent with the results presented above, which showed that SVP-B5 could promote the recovery of hematopoietic system.

During the radiation-induced damage and repair process, the ataxia telangiectasia mutated (ATM) gene plays a critical role in the signal transduction pathway related to DNA double- strand breaks (DSBs). DSBs stimulate ATM phosphorylation within a few minutes, and that effect can last for several hours^16^,^17^. When sensitive proteins detect DSBs, the earliest response of ATM and DNA-protein kinase (DNA-PK) is to phosphorylate histone variant H2AX at Ser 139, generating γ-H2AX^18^. Since each γ-H2AX focus corresponds to one DSB, the γ-H2AX detection has become the gold standard for detecting DSBs^19^. With 14d after TBI, the intranuclear γH2AX levels in mice increased significantly compared with those before TBI, demonstrating that TBI can result in DNA damage. However, after SVP-B5 treatment, the γH2AX levels decreased significantly at 14d after TBI, suggesting that SVP-B5 can reduce the incidence of DSBs or facilitate DNA repair.

Cyclin-dependent kinase inhibitor (CDKI) is a negative regulator of the cell cycle. It leads to cell cycle arrest by inhibiting cyclin-dependent kinase CDK activity, thereby blocking cell proliferation. Both p16 and p21 are members of the CDKI family, and activated p16 and p21 are related to cell cycle arrest. At the same time, the body will initiate the DNA damage repair process. When detecting damaged DNA that is severe and difficult to repair, the DNA damage repair system will induce cell death, mitotic catastrophe, chronic persistent DNA damage repair signals, or senescence^20^, and this persistent DNA damage repair process can prevent the damage from spreading to the next generation of cells. The p16 gene is a recently discovered regulatory gene related to aging. In the aging-related cellular stress responses, activation of the p16 gene can lead to the impairment of the self-renewal capacity of some tissues such as stem cells, while elimination or suppression of p16 gene expression may promote the self-renewal capacity of cells and enhance cell survival^21^. The p21 protein can regulate many cellular processes, including cell cycle arrest, DNA replication, DNA repair and cell differentiation^22^,^23^,^24^. When DNA is injured, p21 can trigger G_1_-phase arrest as a tumor suppressor gene, which may provide time for DNA repair^25^. We speculated from the above results that DNA damage was severe at 7d after TBI and the increased p16 and p21 levels may promote cell cycle arrest and provide more time for DNA repair. At 14d after TBI, SVP-B5 treatment reduced the overall expression levels of p16 and p21, which may have benefited the entry of cells into the cell cycle and promoted the self-renewal, thereby promoting hematopoietic recovery.

## Conclusions

SVP-B5 could effectively reduce ROS levels in the BMNCs of mice after TBI, and alleviate DNA damage. The p16 and p21 expression levels declined after the initial rise after TBI, which might help promote DNA repair in BMNCs and the subsequent entry of cells into the cell cycle and cell proliferation, restored the bone marrow hematopoietic function after irradiation, and increased the survival rate of mice after TBI. The radiation-protective effects of SVP-B5 might be related to the ROS-p16/p21 pathway. We will focus on the molecular target of SVP-B5 in our future research to confirm the in radiation protection.

## Methods

### Mice

Male C57BL/6 mice were purchased from the Guangdong Experimental Animal Center [animal quality certification number: SCXK (Guangdong) 2008–0002] and raised at the Animal Center of Guangzhou Medical College, with 5 mice/cage. All mice were 10 to 12 weeks old and their average body weight was 22 ± 2 g. They were housed in specific-pathogen-free (SPF) conditions and the room temperature was maintained at 24–25 °C. Sterilized food and drinking water were provided. All experimental protocols were conducted in line with generally accepted ethical principles and approved by the Research Ethic Committee of Guangzhou Medical University. The study was performed in accordance with the principals of the Declaration of Helsinki.

### Establishment of animal models of radiation injury

The radiation doses for the experiment were based on our previous studies. The lowest radiation dose which resulted in 100% survival was 6Gy (6 M high-energy linear accelerators, SIMENS PRIMUS) and the highest dose which resulted in 100% lethality was 7.5Gy. Depending on the experiment, C57BL/6 mice were subjected to TBI using 7.5Gy or 6Gy at 300 cGy/min, respectively. The mice were raised in an SPF environment after TBI and sterile food and drinking water were provided.

### Effects of SVP-B5 on the survival of mice exposed to TBI

SVP-B5 was purified by our lab. Our previous acute toxicity test showed that the toxicity of SVP-B5 was low (Its LD_50_ was 0.105 mg/kg), and 1/40 LD_50_ (2.625 μg/kg) could effectively extend the life of mice after TBI. C57 BL/6 mice undergoing 7.5Gy X-ray irradiation were randomly divided into the radiation control group (IR^+^ group) and the SVP-B5 treatment group (IR^+^+SVP-B5 group) to receive SVP-B5 (2.625 μg/kg). There were 15 mice in each group. Saline or SVP-B5 was injected intraperitoneally at 2 h before irradiation and once every other day after irradiation until day 14 after TBI. The experiment was repeated three times. The mice were weighed every day after TBI, and the hair, stool, and mental status were observed. Their symptoms, physical signs, number of deaths, and time of death were recorded until day 30 after irradiation.

### Effects of SVP-B5 on the number of peripheral blood cells in mice exposed to TBI

C57BL/6 mice undergoing 6Gy X-ray irradiation (No death was observed in either group with this radiation dose) were divided into the IR^+^ and IR^+^+SVP-B5 groups and the SVP-B5 was administered in the same manner as described above. Blood samples were collected from the inner canthus before TBI at 5d, 7d, 14d, and 28d after TBI. EDTA was added as an anticoagulant and routine blood tests were performed using an F-820 animal hematology analyzer (Sysmex, Japan) within 1 hour after sample collection. The peripheral blood white blood cell count, neutrophil count, hemoglobin level, and platelet count were recorded.

### Effects of SVP-B5 on the BMNCs of irradiated mice

C57BL/6 mice undergoing 6.0Gy X-ray irradiation were divided into the IR^+^ and IR^+^+SVP-B5 groups. The drug administration method was the same as described above. In each group, five mice were euthanized with CO_2_ at each time point after TBI. The bone marrow cells were flushed out using IMDM (Gibco, USA) containing 3% FBS (Gibco, USA). Red blood lysis buffer (eBioscience, USA) was added to induce lysis of red blood cells. The experiment was repeated three times. Viable cell counts were measured using trypan blue staining to obtain the number of BMNCs.

### Effect of SVP-B5 on the bone marrow colony-forming unit (CFU) of irradiated mice

BMNCs in the IR^−^ (normal control), IR^+^ and IR^+^+SVP-B5 (6Gy or 7.5Gy) groups were collected after irradiation, cells were rinsed with IMDM medium containing 3% FBS and resuspended. The cells were seeded in methocult® medium M3231 (Stem Cell Technologies, USA), at a seeding density of 2 × 10^5^ cells/mL. In the IR^+^+SVP-B5 group, SVP-B5 0.5 μg/mL was added into the culture system. Equal amounts of saline were added in the IR^+^ and the IR^−^ groups. The cells were placed in 24-well plates and three duplicate wells were set up for each group. The cells were cultured in an incubator with 95% air/5% CO_2_ (volume fraction) and saturated humidity at 37 °C. After 14 days, the cells were observed under an inverted microscope to count the CFUs. A CFU was defined as a cell cluster with 50 or more cells. The experiment was repeated three times.

### Effect of SVP-B5 on ROS levels of the BMNCs in mice exposed to TBI

C57BL/6 mice undergoing 6.0Gy X-ray irradiation were divided into the IR^+^ and IR^+^+SVP-B5 groups. The drug administration method was the same as described above. An additional healthy mice were included in the non- radiation control group (IR^−^ group) and IR^−^+SVP-B5 group. Intraperitoneal injection of saline and SVP-B5 was performed. BMNCs were collected 2 h after intraperitoneal injection. BMNCs in the IR^+^ group and IR^+^+SVP-B5 group were collected at 7d and 14d after TBI and resuspended in PBS (Beyotime, China) containing 5 mM glucose, 1 mM CaCl_2_, 0.5 mM MgSO_4_, and 5 mg/mL bovine serum albumin. The Reactive Oxygen Species Assay Kit (Beyotime, China) was used and the kit instructions were followed. BMNCs (1 × 10^6^/mL) were incubated with diluted DCFH-DA (at a final concentration of 10 μmol/L) in the dark at 37 °C for 20 min. In the positive control wells, Rousp was added as a positive control. The cells were rinsed with PBS 3 times after the incubation to fully eliminate non-internalized DCFH-DA. Flow cytometry (Becton-Dickinson, USA) was used to detect the fluorescence intensity of DCF in the BMNCs to assess the ROS levels (at the 488-nm excitation wavelength and the 524-nm emission wavelength).

### Effect of SVP-B5 on TBI-induced DNA damage in mouse BMNCs

BMNCs in IR^−^ group and IR^−^+SVP-B5 group were collected 2 h after injection of saline or SVP-B5, IR^+^ group and IR^+^+SVP-B5 group were treated as described above and B MNCS were collected at 7d and 14d after TBI. The BMNCs were fixed with 100% ethanol for 5 min. The samples were centrifuged and the cells were incubated with 0.1% PBS-Tween for 20 min and centrifuged. After washing with 0.1% PBS-Tween, a mixture of 10% goat serum/PBS/0.3 M glycine was added and the sample was mixed well and placed at 22 °C for 30 min. The non-specific reaction between proteins was blocked. The sample was washed and a sample of 100 μl γH2AX antibody (Abcam, USA)diluted 1:500 was added, and incubated at 22 °C for 30 min. The sample was washed and centrifuged and the supernatant was discarded. The pellets were treated with 100 μl secondary antibody (DyLight® 488 goat anti-mouse IgG) (H+L) (Abcam, USA) diluted 1:500 and incubated at 22 °C for 30 min, washed and centrifuged. Subsequently, 0.5 ml 0.1% PBS-Tween was added, and the γH2AX level was detected by flow cytometry at the FITC wavelength.

### The relative expressions of p16^Ink4a^ and p^21Cip1/Waf1^ in BMNCs

BMNCs in IR^−^ group and IR^−^+SVP-B5 group were collected 2 h after injection of saline or SVP-B5, IR^+^ group and IR^+^+SVP-B5 group were treated as described above and BMNCS were collected at 7d and 14d after TBI and RNA was extracted using the guanidinium isothiocyanate method (TRIZOL kit) (Invitrogen, USA). The absorbance at 260 nm and 280 nm was detected using a UV spectrophotometer to determine RNA yield, and the quality of RNA was observed using a gel imager. RevertAidTM First Strand cDNA Synthesis Kit, ddH_2_O, and 10 μM primer solutions (Fermentas, China) were used to synthesize cDNA. Syber Green I GoTaq qPCR Master Mix (Promega, USA) and SYBR Green Real-time PCR Master Mix (TOYOBO, Japan) were used and p16^INK4a^ and p21^Cip1/Waf1^ mRNA expressions were detected by real-time quantitative PCR using the ABI 7300 PCR System (Applied Biosystems, USA). The matching SDS v1.3.2 software with the PCR instrument was used for analyzing the relative expressions of p16^INK4a^ mRNA, p21^Cip1/Waf1^ mRNA, and internal reference gene GAPDH mRNA. The gene-specific primers were as follows: P21^Cip1/waf1^ (forward: 5′-AATCCTGGTGATGTCCGACC-3′; reverse: 3′-AAAGTTCCACCGTTCTCGG-5′); p16^Ink4a^(forward: 5′-CGGTCGTACCCCGATTCAG-3′; reverse:3′-GCACCGTAGTTGAGCAGAAGAG-5′); GAPDH(forward: 5′-TGAAGGTCGGTGTGAACGGATTTGGC 3′; reverse:3′-CATGTAGGCCATGAGGTCCACCAC-5′). The reaction conditions consisted of pre-denaturation at 95 °C for 60 s, followed by 40 cycles of amplification under the following conditions: 95 °C for 15 s, 60 °C for 15 s, 72 °C for 30 s, and 95 °C for 60 s.

### Statistical analysis

SPSS 15.0 statistical software (Version15.0; SPSS Inc., Chicago, IL, USA) was applied for statistical analysis. Differences of the mean values between groups were analyzed with t-test or analysis of variance. Data from repeated measurements were compared using repeated measure analysis of variance. Counting data are presented with rate or composition ratio and the differences between groups were compared with chi-square test. The K-M method and life table method were used to study the survival of mice. The log-rank test was used to compare the survival rates of different groups. P ≤ 0.05 was considered statistically different.

## Additional Information

**How to cite this article**: Wang, C. *et al.* Effects of Scorpion venom peptide B5 on hematopoietic recovery in irradiated mice and the primary mechanisms. *Sci. Rep.* **5**, 15363; doi: 10.1038/srep15363 (2015).

## Figures and Tables

**Table 1:** Effects of SVP-B5 on the survival of mice after irradiation.

Groups	Average survival time (d)	Median survival time (d)	Long-term survival rate (%)
IR^+^ group	6.67	5.0	0.0
IR^+^+SVP-B5 group	14.87*	10.0*	40.0*

Mice underwent intraperitoneal injection of SVP-B5 (2.63 μg/kg)(IR^+^+SVP-B5 group) or equal amounts of saline (IR^+^ group) 2 h before TBI and once every other day after TBI (7.5Gy) until day 14. The survival rate of mice was observed. The log-rank test showed that χ^2^ = 11.23 *P = 0.000; *P < 0.05 vs. IR^+^ group.
